# Modulation of Synaptic Plasticity by Stress Hormone Associates with Plastic Alteration of Synaptic NMDA Receptor in the Adult Hippocampus

**DOI:** 10.1371/journal.pone.0027215

**Published:** 2011-11-01

**Authors:** Yiu Chung Tse, Rosemary C. Bagot, Juliana A. Hutter, Alice S. Wong, Tak Pan Wong

**Affiliations:** 1 Neuroscience Division, Douglas Mental Health University Institute, McGill University, Montreal, Quebec, Canada; 2 Department of Psychiatry, McGill University, Montreal, Quebec, Canada; 3 Department of Pharmacology and Therapeutics, McGill University, Montreal, Quebec, Canada; Institute for Interdisciplinary Neuroscience, France

## Abstract

Stress exerts a profound impact on learning and memory, in part, through the actions of adrenal corticosterone (CORT) on synaptic plasticity, a cellular model of learning and memory. Increasing findings suggest that CORT exerts its impact on synaptic plasticity by altering the functional properties of glutamate receptors, which include changes in the motility and function of α-amino-3-hydroxy-5-methylisoxazole-4-propionic acid subtype of glutamate receptor (AMPAR) that are responsible for the expression of synaptic plasticity. Here we provide evidence that CORT could also regulate synaptic plasticity by modulating the function of synaptic N-methyl-D-aspartate receptors (NMDARs), which mediate the induction of synaptic plasticity. We found that stress level CORT applied to adult rat hippocampal slices potentiated evoked NMDAR-mediated synaptic responses within 30 min. Surprisingly, following this fast-onset change, we observed a slow-onset (>1 hour after termination of CORT exposure) increase in synaptic expression of GluN2A-containing NMDARs. To investigate the consequences of the distinct fast- and slow-onset modulation of NMDARs for synaptic plasticity, we examined the formation of long-term potentiation (LTP) and long-term depression (LTD) within relevant time windows. Paralleling the increased NMDAR function, both LTP and LTD were facilitated during CORT treatment. However, 1–2 hours after CORT treatment when synaptic expression of GluN2A-containing NMDARs is increased, bidirectional plasticity was no longer facilitated. Our findings reveal the remarkable plasticity of NMDARs in the adult hippocampus in response to CORT. CORT-mediated slow-onset increase in GluN2A in hippocampal synapses could be a homeostatic mechanism to normalize synaptic plasticity following fast-onset stress-induced facilitation.

## Introduction

Corticosterone (CORT) is a stress hormone that mediates a diverse array of physiological functions to facilitate adaptation to homeostatic challenges [1]. Perhaps the best known neurological effect of CORT is as a potent modulator of hippocampal learning and memory [2]–[5], which is related to plastic changes in the efficacy of hippocampal synapses in the form of long-term potentiation (LTP) and long-term depression (LTD) [6], [7]. A wealth of evidence suggests that bidirectional hippocampal synaptic plasticity can be either facilitated [8], [9] or suppressed by CORT [10], [11]. Since synaptic plasticity is mediated by activation of glutamate receptors, CORT could regulate synaptic plasticity through altering glutamate receptor function. Indeed, a brief treatment of CORT enhances the synaptic current, surface expression, and motility of α-amino-3-hydroxy-5-methylisoxazole-4-propionic acid subtype of glutamate receptor (AMPAR [12]–[15]), which are instrumental for the expression of synaptic plasticity. Further studies on the effects of CORT on glutamate receptors may reveal mechanisms of how this stress hormone regulates synaptic plasticity.

Given the primary role of N-methyl-D-aspartate receptors (NMDARs) in the induction of bidirectional synaptic plasticity [16], [17], CORT-induced changes in NMDAR function may also substantially impact synaptic plasticity. CORT has variously been shown to increase [18] and decrease [19], [20] NMDAR function in young hippocampal tissue (<1 month). However, questions remain concerning the role of altered NMDAR function in CORT-induced regulation of synaptic plasticity. CORT modulates NMDAR function in young hippocampal tissue, however current evidence suggests that NMDAR becomes less plastic after early brain development [21]. Although prolonged exposure to stress [22], [23] and CORT [24]–[26] or corticosteroid receptor agonists [27] have been shown to alter the expression of NMDAR subunits in the adult brain, whether CORT acutely regulates NMDAR function in adult tissue is still unknown. Since most studies examining effects of stress and/or CORT in synaptic plasticity were performed in the adult hippocampus [28]–[31], in order to probe the potential contribution of CORT-induced changes in NMDAR to CORT regulation of bidirectional synaptic plasticity it is essential to first examine the effects of acute CORT on NMDAR function in mature hippocampal tissue. Another pertinent question is whether CORT acutely affects the GluN2 subunit composition of NMDAR. NMDARs are multimeric ionotropic glutamate receptors, the majority of which consist of GluN1 and GluN2 subunits [32]. The discovery that antagonists against GluN2A- and GluN2B-containing receptors specifically inhibit LTP and LTD formation, respectively [33], revealed the critical role of NMDAR GluN2 subunit composition in regulating the direction of synaptic plasticity (for review, see [34], [35]). Notably, exposure to repeated or long-term stressors alters the GluN2 composition of NMDAR [36]–[39]. Critically, whether acute CORT exposure, which induces changes in hippocampal plasticity [11], [40], [41], is sufficient to alter GluN2 subunits in the adult hippocampus remains unknown.

We used an adult brain slice model (3-month-old) to determine if CORT alters the strength and GluN2 subunit composition of NMDAR, and if these CORT-induced changes relate to the regulation of bidirectional synaptic plasticity by this stress hormone.

## Results

### CORT enhances synaptic NMDAR function in the adult hippocampus

**Figure 1:**
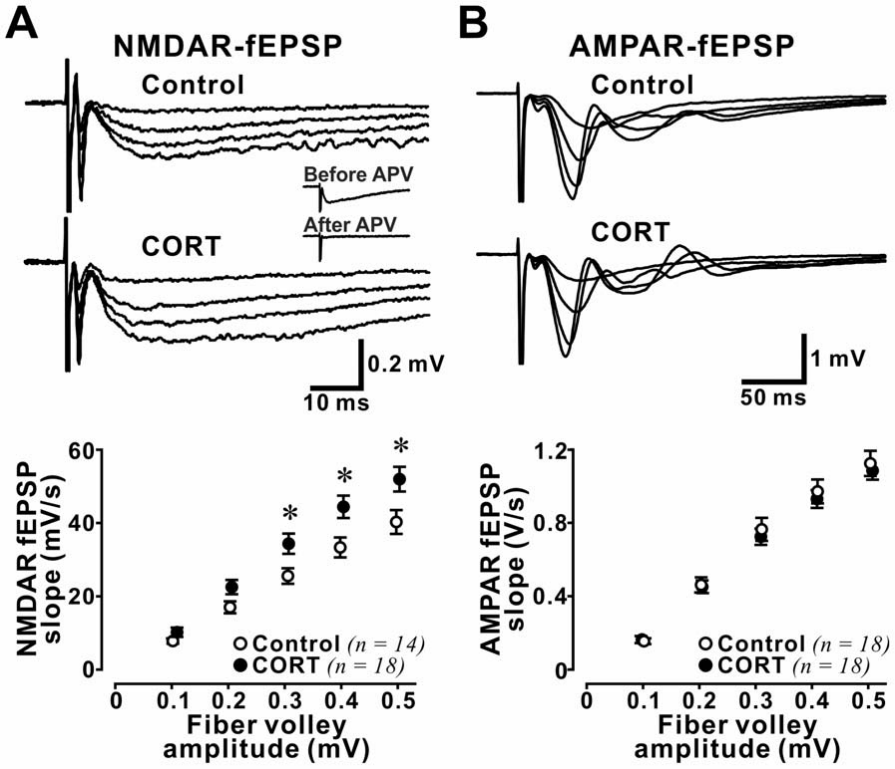
Stress level CORT increased NMDAR-mediated fEPSP in the adult hippocampus. (**A**) *Upper panel:* representative average traces of evoked field excitatory postsynaptic potentials (fEPSP) mediated by NMDAR. Strength of stimulation was adjusted to produce fiber volley sizes ranging from 0.1 to 0.5 mV. *Inset:* Traces of NMDAR-mediated fEPSPs measured before and after APV treatment (50 µM). Note that fEPSP but not the fiber volley and stimulation artefact was abolished by APV. *Lower panel:* Plots of the relationship between fiber volley size and fEPSP slope in both control and CORT-treated hippocampal slices. Note that CORT significantly increased NMDAR-fEPSP at fiber volley size of 0.3-0.5 mV. * p<0.05: post-hoc *Fisher*'*s test* after *ANOVA*. (**B**) *Upper panel:* representative average traces of AMPAR-fEPSP. *Lower panel:* Plots of the relationship between fiber volley size and AMPAR-fEPSP slope. No difference was observed between these parameters in control and CORT-treated slices.

We first examined the impact of stress level CORT (100 nM) on evoked NMDAR-fEPSP. Previous microdialysis studies [42] indicate that 100 nM CORT is in the range of CORT levels measured *in vivo* in rat hippocampus shortly after exposure to an intense acute stressor such as forced swimming. Recordings of NMDAR-fEPSP were performed within 20 to 30 min after the beginning of CORT treatment to isolate rapid effects of CORT on synaptic NMDARs. Stimulation intensity was varied to produce fEPSPs with increasing fiber volley size ranging from 0.1 to 0.5 mV to control for the variability of evoked synaptic responses in brain slices (Fig. 1A). We found that CORT significantly increased NMDAR-fEPSP slope in hippocampal synapses (*repeated measures ANOVA*: effect of CORT: *F(1,30)* = 5.60, p = 0.025; effect of fiber volley size: *F(4,120)* = 216.3, p<0.001; interaction between effects of CORT and fiber volley size: *F(4,120)* = 3.88, p = 0.005). Post-hoc *Fisher*'*s test* showed significant increases in NMDAR-fEPSP at fiber volley size of 0.3 mV (p = 0.028), 0.4 mV (p = 0.018), and 0.5 mV (p = 0.029), indicating that in the adult hippocampus, stress level CORT enhances synaptic NMDAR function.

Since CORT enhances action potential-independent AMPAR-mediated synaptic function in hippocampal synapses [13], we asked if CORT also affects synaptic AMPAR function in our preparation. We examined evoked AMPAR-fEPSP in the presence of a NMDAR antagonist APV (50 µM), to prevent contamination of fEPSP by NMDAR activation induced by strong electrical stimulation (Fig 1B). Surprisingly, we found no effect of stress level CORT on AMPAR-mediated postsynaptic responses (*repeated measures ANOVA* analysis: effect of CORT: *F(1,34)* = 0.16, p = 0.695; effect of fiber volley size: *F(4,136)* = 613.0, p<0.001; interaction between effects of CORT and fiber volley size: *F(4,136)* = 0.69, p = 0.602). Since NMDAR blockade abolishes stress-induced modulation of synaptic plasticity [43], blocking NMDAR with APV may have abolished any potential impact of CORT on AMPAR in our preparation. However, when we examined the fEPSP-fiber volley relationship in slices not treated with APV, we similarly observed no change in AMPAR function in CORT-treated slices (*repeated measures ANOVA* analysis: effect of CORT: *F(1,9)* = 0.009, p = 0.927; effect of fiber volley size: *F(4,36)* = 35.3, p<0.001; interaction between effects of CORT and fiber volley size: *F(4,36)* = 0.038, p = 0.997).

### Long-lasting enhancement in NMDAR function in the adult hippocampus after CORT treatment

**Figure 2:**
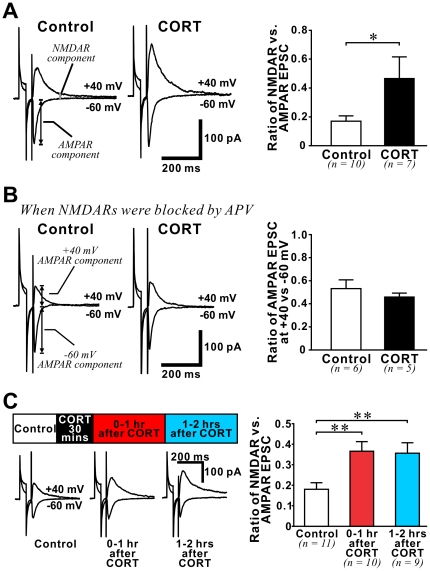
Stress level CORT increased NMDAR/AMPAR ratio of evoked EPSC in the adult hippocampus. (**A**) *Left:* Representative average traces of evoked excitatory postsynaptic currents (EPSCs) recorded from CA1 pyramidal neurons under control condition or during CORT treatment. CA1 neurons were voltage clamped at −60 mV to isolate the AMPAR component (peak of EPSC). The NMDAR component of EPSCs was measured in the same neurons while voltage-clamping them at +40 mV. EPSC amplitude at 150 ms after the stimulating artefact, when AMPAR component has returned to baseline, was used to calculate the NMDAR component. *Right:* histogram summarizes the ratio of NMDAR/AMPAR recorded in control and during CORT slices. CORT significantly increased the NMDAR/AMPAR ratio. * p<0.05: *student*'*s t-test*. (**B**) CORT did not affect the ratio of EPSC amplitude recorded at +40 and −60 mV holding potential when NMDAR was blocked by APV (50 µM). (**C**) *Left:* representative average traces of evoked EPSCs recorded from CA1 pyramidal neurons in control slices and in CORT-treated slices in two time windows: 0–1 hour and 1–2 hours *after CORT* (depicted by a schematic time line above traces). Changes in NMDAR/AMPAR ratio caused by a 30-min CORT treatment at these time windows are summarized in a histogram *(right)*. Note that NMDAR/AMPAR ratio is significantly increased in both post-CORT treatment time windows. ** p<0.01: post-hoc *Fisher*'*s test* after *ANOVA*.

If CORT increases NMDAR- but not AMPAR-mediated synaptic responses, we predicted that the ratio between these two components of synaptic current would be altered by CORT. To investigate this, we recorded evoked excitatory postsynaptic current (EPSC) from individual CA1 pyramidal neurons in whole-cell mode when GABA_A_Rs were blocked by bicuculline (10 µM). We isolated AMPAR- and NMDAR+AMPAR-mediated EPSCs by voltage-clamping neurons at −60 and +40 mV, respectively (Fig. 2A). The NMDAR component was then measured at 150 ms after the stimulation artefact in NMDAR+AMPAR-mediated EPSC, when the AMPAR component has returned to baseline. We found that CORT significantly increased the NMDAR/AMPAR ratio (p = 0.039). As we did not observe significant effects of CORT on either the series resistance of recordings (13.9±1.3 MΩ in control *vs.* 15.3±1.6 MΩ in CORT-treated slices) or the input resistance of recorded neurons (170.5±14.5 MΩ in control *vs.* 154.9±22.7 MΩ in CORT-treated slices), changes in NMDAR/AMPAR ratio in CORT-treated slices cannot be attributed to differences in the quality of voltage clamping between experiments. Finally, when we measured the +40/−60 ratio in the presence of APV (50 µM), we found no change in this ratio between control and CORT-treated slices (Fig. 2B), confirming that the change in +40/−60 ratio after CORT treatment was caused by alteration of NMDAR function only.

To investigate if the CORT-induced increase in NMDAR function is long-lasting we examined the impact of a 30-min stress level CORT treatment on the NMDAR/AMPAR ratio in two post-treatment time windows: *0*–*1 hr* and *1*–*2 hrs after CORT* (Fig. 2C). In both time windows we found that the NMDAR/AMPAR ratio was significantly higher than that in control slices (*ANOVA analysis* (control and 2 CORT-treated groups): *F(2,27) = *6.23, p = 0.006; post-hoc comparison: control (n = 11) *vs. 0*–*1 hr after CORT* (n = 10), p = 0.004; control *vs. 1*–*2 hrs after CORT* (n = 9), p = 0.008).

We next asked if the long lasting increase in NMDAR/AMPAR ratio 1–2 hours after CORT treatment was specifically due to increased NMDAR function, or rather caused by a decrease in AMPAR function at this late time window. We separately examined the fiber-volley/fEPSP ratio of NMDAR- and AMPAR-fEPSP in slices 1–2 hours after CORT. We found that at this late time window, CORT still significantly increased NMDAR-fEPSP slope in hippocampal synapses (*repeated measures ANOVA*: effect of CORT: *F(1,16)* = 5.94, p = 0.027; effect of fiber volley size: *F(4,64)* = 205.4, p<0.001; interaction between effects of CORT and fiber volley size: *F(4,64)* = 3.26, p = 0.017). However, CORT did not affect AMPAR-fEPSP (*repeated measures ANOVA*: effect of CORT: *F(1,10)* = 0.50, p = 0.494; effect of fiber volley size: *F(4,40)* = 94.7, p<0.001; interaction between CORT and fiber volley size: *F(4,40)* = 0.40, p = 0.810). Taken together, our findings illustrate an enduring increase in synaptic NMDAR function in the adult hippocampus after a brief exposure to stress level CORT.

### Mechanisms underlying CORT-induced enhancement of synaptic NMDAR function

**Figure 3:**
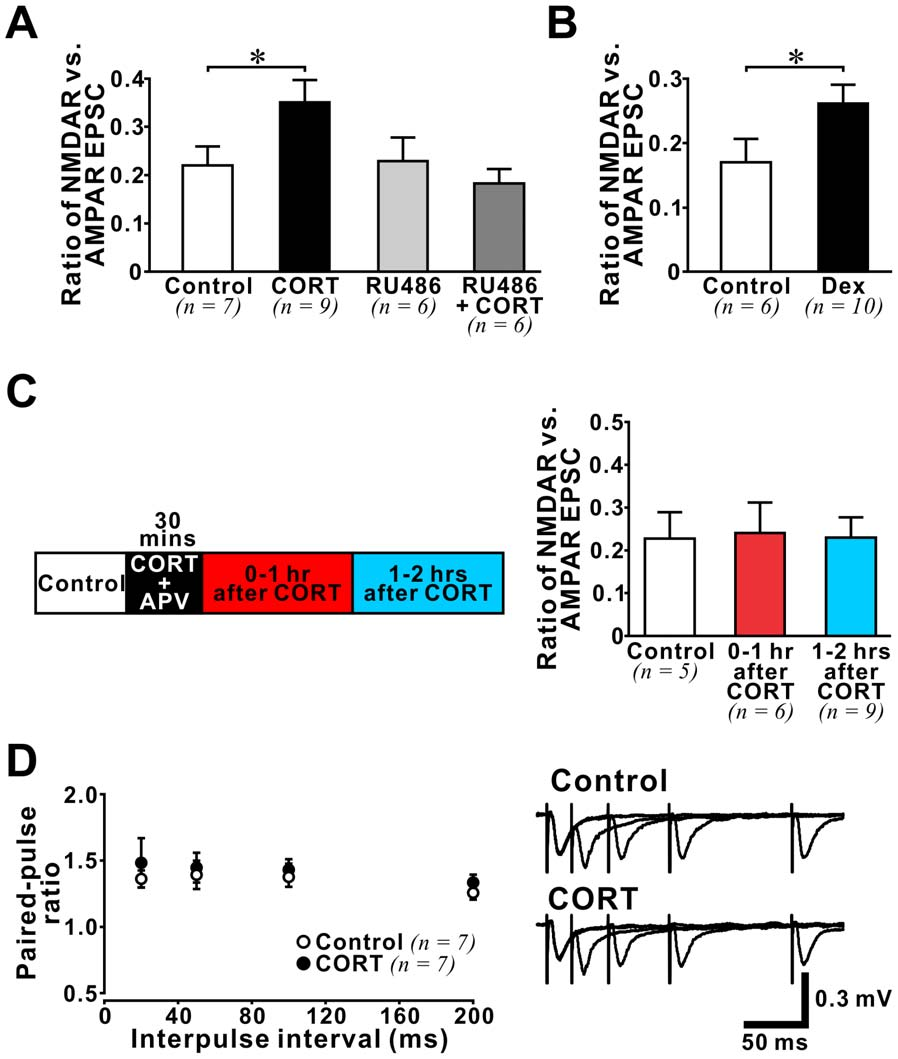
Characteristics of CORT-induced enhancement of NMDAR-mediated synaptic function in the adult hippocampus. (**A**) Histogram shows the NMDAR/AMPAR ratio recorded from slices exposed to one of the following treatments: control (white), CORT (black (100 nM)), GR antagonist RU486 (light grey (500 nM)), and RU486 + CORT (dark grey). Note that only CORT-treated slices exhibited an increase in NMDAR/AMPAR ratio. * p<0.05: post-hoc *Fisher*'*s test* after *ANOVA*. (**B**) Increase in NMDAR/AMPAR ratio was induced by a GR agonist dexamethasone (Dex (200 nM)). * p<0.05: *student*'*s t-test*. (**C**) *Left:* A schematic time line illustrates the time windows for drug treatment (CORT (100 nM) + APV (50 µM)) and recording. Evoked EPSCs were recorded from CA1 pyramidal neurons in control slices and drug-treated slices in two time windows: 0–1 hour and 1–2 hours after CORT treatment. Changes in NMDAR/AMPAR ratio at these time windows were summarized in a histogram *(right)*. Note that CORT did not alter NMDAR/AMPAR ratio when NMDAR was blocked by APV during CORT treatment. (**D**) Plots on the *left* show no difference between paired-pulse ratios recorded from control slices (white circles) and slices during CORT treatment (black circles). Average traces on the *right* are representative examples.

We reasoned that the enhancement of NMDAR function by stress level CORT was likely mediated by the low affinity glucocorticoid receptors (GRs) that are sensitive to high CORT levels [44]. We examined the effect of a GR antagonist RU486 (Cayman chemical) on the CORT-induced increase in NMDAR/AMPAR ratio and found that RU486 (500 nM) abolished the effect of CORT (Fig. 3A). Treating slices with RU486 alone had no effect on NMDAR/AMPAR ratio (*ANOVA* analysis between control, CORT, RU486 alone, and RU486 + CORT groups: *F(3,24) = *3.16, p = 0.043; post-hoc comparison: control *vs.* CORT, p = 0.035; control *vs.* RU486 alone, p = 0.915; control *vs.* RU486 + CORT, p = 0.558). In addition, treating adult brain slices with a GR-specific agonist dexamethasone (Dex, 200 nM), which binds GRs but not the high affinity mineralocorticoid receptors, was sufficient to increase the NMDAR/AMPAR ratio (Fig. 3B; p = 0.046).

The impact of stress on hippocampal synaptic plasticity requires NMDAR activation during stress [43]. To test if CORT-induced increase in synaptic NMDAR current also depends on NMDAR activation, we treated adult brain slices with CORT plus APV (50 µM) for 30 min before measuring the NMDAR/AMPAR ratio of evoked EPSCs in the absence of APV (Fig. 3C). We found that the presence of APV during CORT treatment prevented the change of NMDAR/AMPAR ratio in both *0*–*1 hr* and *1*–*2 hrs after CORT* time windows (*ANOVA* analysis between control, *0*–*1 hr after CORT* and *1*–*2 hrs after CORT*: *F(2,17) = *0.013, p = 0.987). These findings demonstrate that NMDAR activation during CORT treatment is required for the facilitation of synaptic NMDAR current.

We next examined if an increase in presynaptic release of glutamate could be responsible for the enhancement of synaptic NMDAR function after CORT treatment. During CORT treatment we measured paired-pulse ratio of fEPSPs at different interpulse intervals (20–200 ms), a commonly used functional assay of presynaptic effects (Fig. 3D). We found no effect of CORT on the paired-pulse ratio at any time interval tested, suggesting that CORT did not affect presynaptic glutamate release in the adult hippocampus.

**Figure 4:**
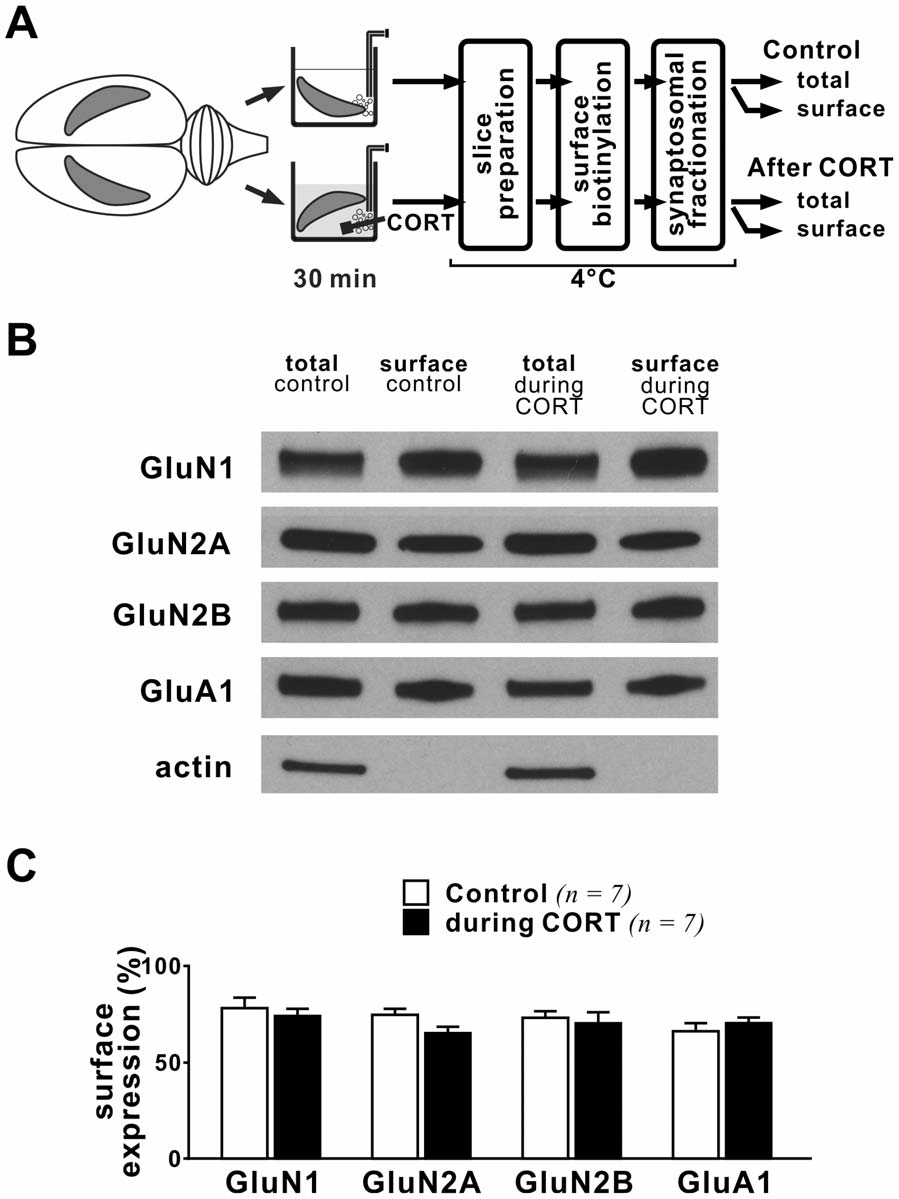
Trafficking of glutamate receptors in adult hippocampal synapses during CORT treatment. (**A**) Schematic diagram shows procedures for drug treatment and tissue labeling. The two hippocampi from each rat were either incubated with aCSF (control) or CORT (100 nM, 30 min). At the end of CORT treatment, these hippocampi were cut into slices and labeled as described in *Methods*. Note that all labeling and purification steps were performed at 4°C. Surface and total proteins from control and CORT-treated hippocampi of each rat were compared by western blotting. (**B**) Representative blots of different glutamate receptor subunits and actin in synaptosomal membranes from control and CORT-treated hippocampi of the same rat. (**C**) Histogram summarizes the surface expression of glutamate receptors (ratio of surface/total) in control and CORT-treated hippocampi. Note that the surface expression of glutamate receptors was not altered during CORT treatment.

Previous findings obtained from immature prefrontal cortical slices [45] or cultures [46] suggest that insertion of NMDAR into synapses could be responsible for the enhanced NMDAR function after CORT treatment. To determine if NMDAR trafficking underlies the increased synaptic NMDAR function during CORT treatment in adult hippocampus, we used a modified biotinylation assay to examine the surface expression of different subunits of NMDAR in adult brain slices immediately after a 30-min long CORT treatment (Fig. 4). To better estimate changes of NMDAR in synapses, we compared the surface expression of NMDAR in the synaptosomal fraction of hippocampal lysates. Although we observed potentiation of synaptic NMDAR function during CORT treatment (Fig. 1, 2), we did not observe corresponding changes in the surface expression of GluN1, GluN2A or GluN2B subunits (Fig. 4B, C). Nor did we observe a change in the surface expression of GluA1, an AMPAR subunit, during CORT treatment. Our findings suggest that an increase in surface expression of NMDAR is not responsible for the initial enhancement of synaptic NMDAR current in adult hippocampal slices during CORT treatment.

**Figure 5:**
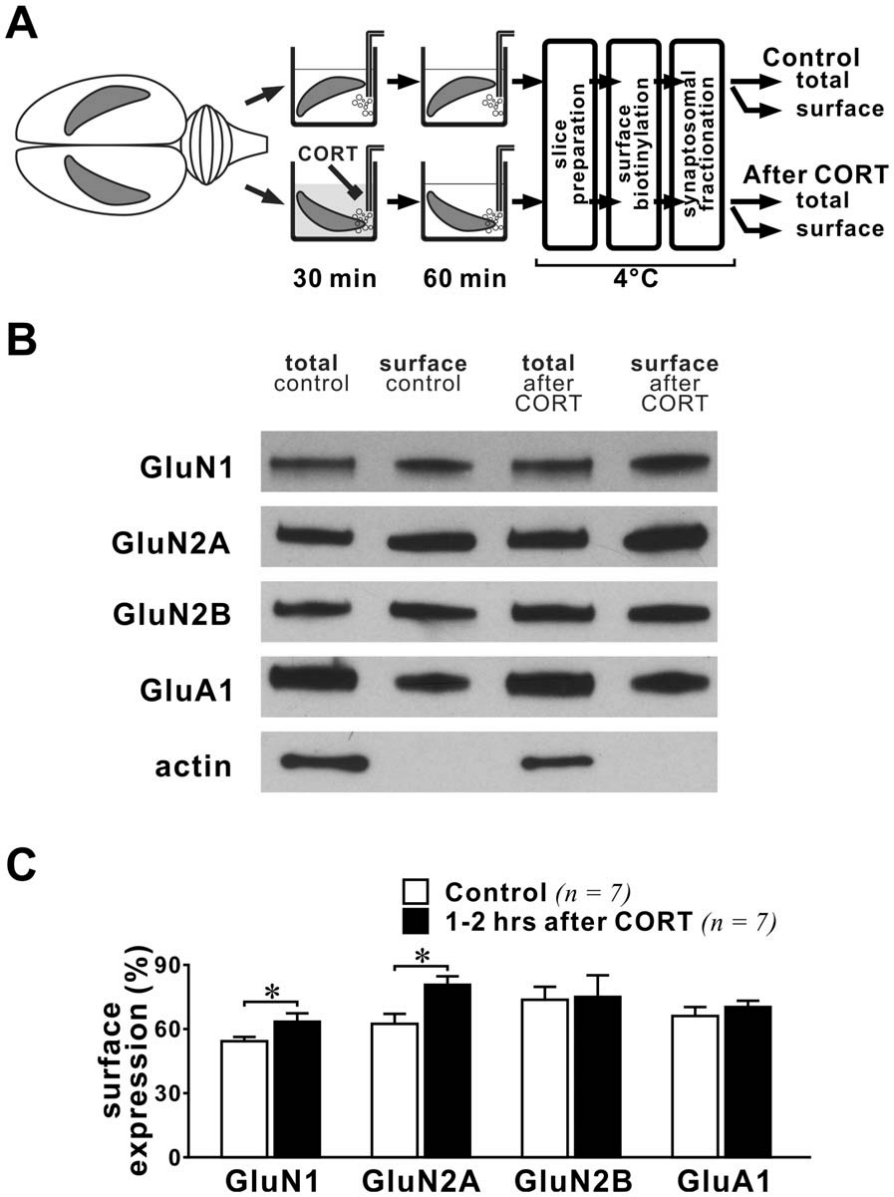
Trafficking of glutamate receptors in adult hippocampal synapses 1–2 hours after CORT treatment. (**A**) Schematic diagram shows procedures for drug treatment and tissue labeling. Note that unlike *Figure 4*, hippocampi were incubated in aCSF for 1–2 hours after CORT treatment before labeling. (**B**) Representative blots of different glutamate receptor subunits and actin in synaptosomal membranes from control and CORT-treated hippocampi of the same rat. (**C**) Histogram summarizes the surface expression of glutamate receptors (ratio of surface/total) in control and CORT-treated hippocampi. Note that the surface expression of both GluN1 and GluN2A was increased by CORT at this time window. * p<0.05; *paired student*'*s t-test (control vs. CORT-treated hippocampi from the same rat)*.

Increased surface expression of NMDAR after acute stress or CORT treatment in young prefrontal cortical tissue was observed 1–4 hours after stress [45], thus we examined if there is a slow-onset increase in NMDAR surface expression in adult hippocampal slices after CORT treatment. We compared the surface expression of different subunits of NMDAR between control and slices exposed to CORT (30 min, 100 nM) 1–2 hours prior to homogenization (Fig. 5). We found that the percent surface expression of GluN1 (p = 0.013) and GluN2A subunits in synaptosomal membranes was significantly increased (p = 0.011). However, we observed no changes in the surface expression of GluN2B or GluA1 (an AMPAR subunit). These data strongly suggest that CORT induces a slow-onset increase in the surface expression of NMDAR in hippocampal synapses.

### CORT induces a slow-onset change in GluN2 composition

**Figure 6:**
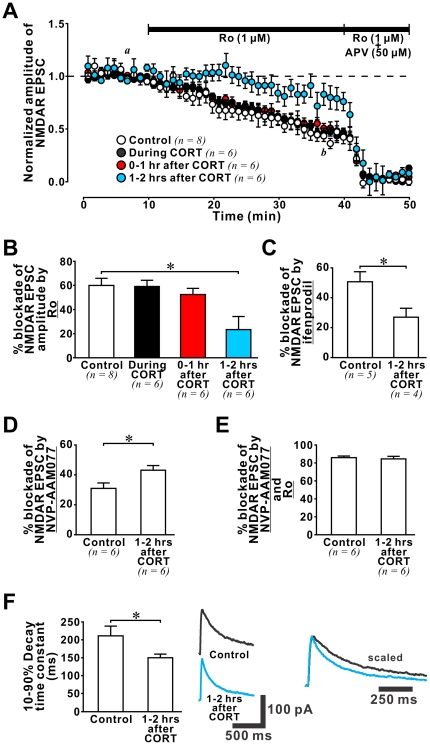
CORT triggered a slow-onset increase in GluN2A/GluN2B ratio. (**A**) Plots of NMDAR-EPSC amplitude from control and CORT-treated slices against time show decreased EPSC amplitude after sequential application of Ro (1 µM; for 30 min) and a mixture of Ro and a subunit non-selective NMDAR antagonist APV (50 µM; for 10 min). The dashed line represents 100% normalized NMDAR-EPSC amplitude. Note that Ro produced significantly smaller blockade of NMDAR-EPSCs recorded in *1*–*2 hrs after CORT* slices (blue circles). (**B**) Histogram summarizes the percentage blockade of Ro in slices from 4 different groups. * p<0.05: post-hoc *Fisher*'*s test* after *ANOVA*. (**C**) Histogram summarizes the percentage blockade by ifenprodil (1 µM), another GluN2B antagonist, in NMDAR-EPSCs recorded in control and *1*–*2 hrs after CORT* slices. Note that ifenprodil produced significantly smaller blockade of NMDAR-EPSCs recorded in *1*–*2 hrs after CORT* slices. * p<0.05: *student*'*s t-test*. (**D**) Histogram summarizes the percentage blockade by NVP-AAM077 (50 nM), a GluN2A antagonist, in NMDAR-EPSCs recorded in control and *1*–*2 hrs after CORT* slices. Note that NVP-AAM077 produced significantly greater blockade of NMDAR-EPSCs recorded in *1*–*2 hrs after CORT* slices. * p<0.05: *student*'*s t-test*. (**E**) Histogram summarizes the percentage blockade by a cocktail of NVP-AAM077 and Ro in NMDAR-EPSCs recorded in control and *1*–*2 hrs after CORT* slices. Note the similar blockade of NMDAR-EPSCs in control and *1*–*2 hrs after CORT* slices. (**F**) *Left:* Histogram summarizes the 10–90% decay time constant of evoked NMDAR-EPSCs recorded in control and *1*–*2 hrs after CORT* slices. Note that the decay time constant of NMDAR-EPSC recorded from *1*–*2 hrs after CORT* slices was significantly smaller than that from control slices. * p<0.05: *student*'*s t-test*. *Middle:* Representative traces of evoked NMDAR-EPSC recorded from control and *1*–*2 hrs after CORT* slices. *Right:* Traces were peak-scaled to illustrate the faster decay property of NMDAR-EPSCs in *1*–*2 hrs after CORT* slices.

Since we observed an increase in the surface expression of GluN2A but not GluN2B subunit after CORT treatment, our findings suggest that the functional contribution of GluN2A- and GluN2B-containing receptors may be altered by CORT. Specifically, an increase in the functional contribution of GluN2A-containing receptors should be observable 1–2 hours after CORT treatment. To test this, we examined the effect of CORT on the functional contribution of GluN2A- and GluN2B-containing NMDARs in hippocampal synapses using subunit-specific antagonists, such as a GluN2B antagonist Ro25-6981 (Ro, 1 µM, [47]) to estimate the GluN2B content of evoked NMDAR-mediated EPSCs (Fig. 6A). Consistent with results of our surface biotinylation experiments, we found no change in GluN2B content during CORT treatment. We then examined the GluN2B content of synaptic NMDAR currents 0–1 hour (*0*–*1 hr after CORT*) and 1–2 hours (*1*–*2 hrs after CORT*) after the end of CORT treatment. While we found no significant change in the GluN2B content of synaptic NMDAR currents recorded 0–1 hour after CORT treatment, we observed a significant decrease in GluN2B content 1–2 hours after CORT treatment (Fig. 6B; *ANOVA* analysis (control and 3 CORT-treated groups): *F(3,22)* = 5.57, p = 0.005; post-hoc comparison: control *vs. during CORT*: p = 0.906; control *vs. 0*–*1 hr after CORT*: p = 0.473; control *vs. 1*–*2 hrs after CORT*: p = 0.001). This slow-onset decrease in GluN2B content was confirmed by experiments using another GluN2B antagonist ifenprodil (1 µM; control vs. CORT: p = 0.036; Fig. 6C). Taken together, our electrophysiological findings reveal a reduced functional contribution of GluN2B-containing receptors in adult hippocampal synapses after CORT treatment.

Next, we tested if CORT increases the GluN2A content in glutamate synapses 1–2 hours after CORT treatment. We employed NVP-AAM077 at a concentration (50 nM) that has been shown to selectively block GluN2A-containing receptors in rats [48]. As expected, we found that the blockade of evoked EPSCs by NVP-AAM077 in control slices was significantly decreased relative to that in *1*–*2 hrs after CORT* slices (Fig. 6D; p = 0.026). Note that in adult hippocampal synapses, there is a small but functionally significant contribution of non-GluN2A/GluN2B-containing receptors, such as GluN2D-containing receptors [49], [50]. To determine if CORT alters the functional contribution of non-GluN2A/GluN2B receptors in adult hippocampal synapses, we compared the combined blockade of synaptic NMDAR current by a cocktail of NVP-AAM077 and Ro between control and *1*–*2 hrs after CORT* slices and found no differences (Fig. 6E), suggesting that CORT exerted no long-term effect on non-GluN2A/GluN2B-containing receptors in adult hippocampal synapses.

Since GluN2A-containing receptors display faster decay kinetics than GluN2B-containing receptors [32], we anticipated the decay time constant of synaptic NMDAR current would be shortened by CORT. Consistent with this prediction, we found that the 10–90% decay time constant of synaptic NMDAR currents was significantly reduced in neurons from *1*–*2 hrs after CORT* slices (Fig. 6F, control: 211.6±26.6 ms *vs. 1*–*2 hrs after CORT:* 150.4±9.6 ms, p = 0.039). Taken together, these electrophysiological data strongly suggest that CORT triggers a slow-onset (1 hour after CORT treatment) increase in the GluN2A/GluN2B ratio of synaptic NMDAR current.

### CORT-induced modulation of synaptic NMDAR and synaptic plasticity in the adult hippocampus

**Figure 7:**
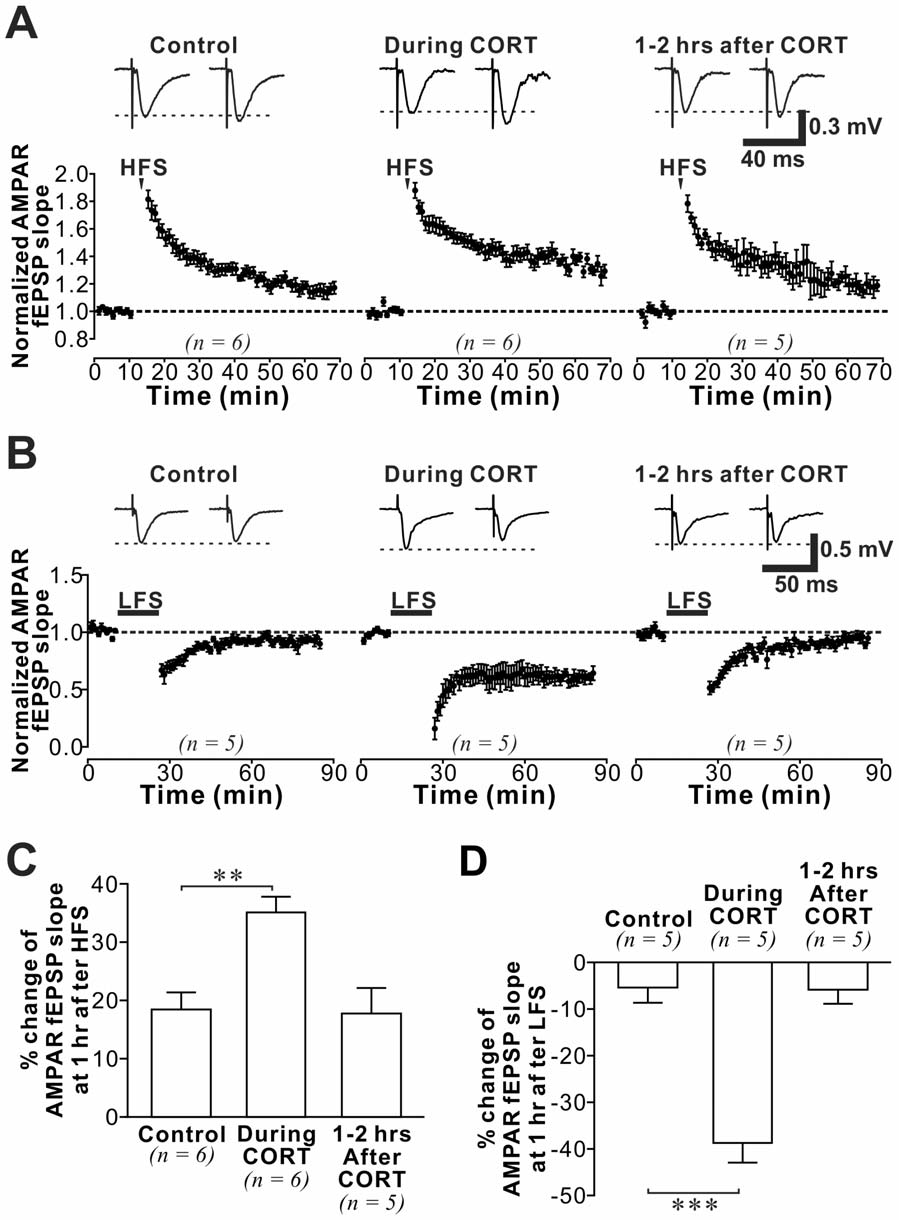
CORT-induced time-dependent regulation of bidirectional plasticity. (**A**) Plots of AMPAR-mediated fEPSP slope against time from data recorded in control, during CORT, and 1-2 hours after CORT. LTP was induced by high frequency stimulation (HFS; 100 Hz, 100 pulses). Representative traces were obtained before (*left*) and 55 min after HFS (*right*). (**B**) Plots of AMPAR-mediated fEPSP slope against time from data recorded in control, during CORT, and 1–2 hours after CORT treatment. LTD was induced by low frequency stimulation (LFS; 1 Hz, 900 pulses). (**C**) Histogram summarizes the percentage change of AMPAR-mediated fEPSP slope in control and different CORT-treated groups at 55 min after HFS or LFS. Note that the facilitation of LTP and LTD caused by CORT was abolished 1–2 hours after CORT treatment. Post-hoc *Fisher*'*s test* after *ANOVA*: ** p<0.01, *** p<0.001.

Finally, we reasoned that CORT-induced changes in NMDAR function and subunit composition could affect bidirectional hippocampal plasticity of synaptic AMPAR currents. Thus we examined LTP and LTD in control slices, in slices during CORT treatment when synaptic NMDAR function is facilitated, and in slices pretreated with CORT 1–2 hours prior to plasticity induction when both the strength and subunit composition of synaptic NMDAR are altered (Fig. 7). Compared with control, we found that the percent potentiation of fEPSP during LTP (1 hour after HFS) was significantly stronger during CORT treatment. This confirms that acute CORT treatment facilitates LTP. Interestingly, similar facilitation could not be demonstrated in slices treated with CORT 1–2 hours ago, in which the GluN2A/GluN2B ratio was increased at the time of LTP induction (*ANOVA* analysis (control and 2 CORT-treated groups): *F(2,16)* = 9.09, p = 0.003; post-hoc comparison: control (n = 6) *vs. during CORT*: p = 0.002; control *vs. 1*–*2 hrs after CORT*: p = 0.896). Similar to the CORT-induced enhancement of LTP, we found that LTD was also facilitated during CORT treatment, and yet no facilitation of LTD was observed 1-2 hours after CORT (*ANOVA* analysis (control and 2 CORT-treated groups): *F(2,12)* = 28.62, p<0.001; post-hoc comparison: control *vs. during CORT*: p<0.001; control *vs. 1*–*2 hrs after CORT*: p = 0.930). In summary, CORT induced a transient facilitation of bidirectional hippocampal synaptic plasticity, which returned to control levels when GluN2A/GluN2B ratio was enhanced by CORT.

## Discussion

**Figure 8:**
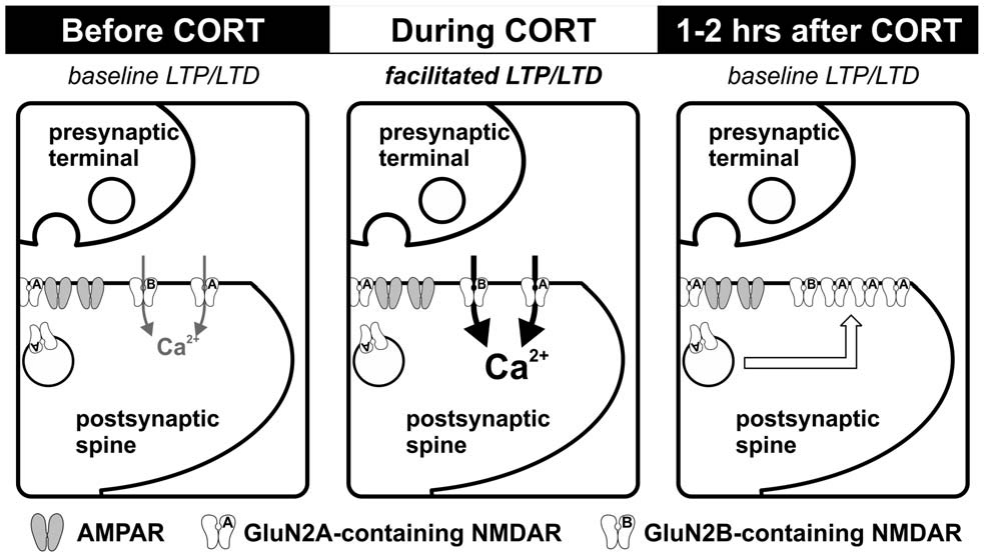
CORT-induced dynamic regulation of synaptic NMDARs in the adult hippocampus. Schematic diagrams summarize the impact of CORT on NMDAR function and synaptic plasticity. Compared with controls (*left*), stress level CORT treatment (100 nM, 30 min) induces a fast-onset increase in synaptic NMDAR function, possibly caused by increased NMDAR channel function or cleft glutamate availability (*center*). Notably, both LTP and LTD are facilitated during CORT treatment. However, 1–2 hours after the end of CORT treatment, we observed enhancement of the surface expression of GluN2A-containing NMDAR (*right*). This slow-onset increase in GluN2A/GluN2B ratio after CORT treatment could have a homeostatic role for normalizing the facilitated synaptic plasticity.

We show that a single, brief CORT exposure dynamically regulates synaptic NMDAR function in the adult hippocampus (Fig. 8). CORT induced a fast-onset increase in NMDAR-mediated synaptic function during a 30-min exposure. This relatively rapid change, which required both GR and NMDAR activation, was not accompanied by alterations in paired-pulse ratio or synaptic expression of NMDARs, suggesting that neither presynaptic changes nor receptor trafficking are responsible for the initial facilitation. CORT also induced a slow-onset increase in GluN2A/GluN2B ratio in hippocampal synapses, detectable one hour after CORT treatment. In parallel with these plastic changes of synaptic NMDARs, we observed facilitation of bidirectional hippocampal synaptic plasticity during CORT treatment. However, CORT-induced facilitation of synaptic plasticity was no longer evident at a time-point when the GluN2A/GluN2B ratio was enhanced (one hour after termination of CORT treatment). These findings strongly suggest that plastic changes in NMDARs represent cellular mechanisms triggered by CORT exposure to regulate bidirectional synaptic plasticity in the adult hippocampus.

We demonstrate that activations of GR and NMDAR during CORT treatment are both required for the enhancement of NMDAR function. Since NMDARs are highly permeable to Ca^2+^ [51], and activation of corticosteroid receptors enhances voltage-gated Ca^2+^ channel functions (VGCC [52], [53]), our findings suggest that a rise of intracellular Ca^2+^ may be necessary for CORT to enhance NMDAR function. In support of this hypothesis, serum- and glucocorticoid-inducible kinase (SGK), a kinase associated with CORT-induced enhancement of glutamatergic transmission in the prefrontal cortex [46], can be activated by intracellular Ca^2+^ [54]. Influx of Ca^2+^ through NMDARs and VGCCs after CORT treatment may activate SGK to enhance NMDAR function. Alternatively, Ca^2+^ influx could also activate other kinases, such as CaMKII [55], PKC [56] and MAP kinases [57] that are potential downstream signals of CORT. Whether these kinases are responsible for CORT-induced regulation of synaptic NMDAR function is not clear.

Previous findings obtained from young cortical tissue suggest that enhancement of NMDAR function 1–4 hours after acute stress or CORT treatment relates to increased surface expression of NMDAR [45], [46]. We found that the surface expression of NMDAR in glutamate synapses was not enhanced during a 30-min CORT treatment. Rather, significant increase in the surface expression of GluN1 and GluN2A subunits in hippocampal synapses was observed only 1–2 hours after CORT treatment. Notably, no change in the synaptic expression of GluN1 subunit was observed in the hippocampus of adult mice subjected to a stressful Morris water maze training session of less than an hour duration [58]. These findings suggest that insertion of NMDAR into adult hippocampal synapses is a slow-onset CORT-induced change. Since synaptic NMDAR function was enhanced at the time window when synaptic expression of NMDAR remained unaltered, our findings further suggest that the fast-onset CORT-induced potentiation of NMDAR synaptic function is caused by mechanisms that are unrelated to receptor trafficking.

Other than receptor trafficking, several mechanisms could be responsible for enhancing NMDAR function during or shortly after CORT exposure. Steroid hormones such as pregnenolone have been shown to directly modulate the gating properties of NMDAR [59]. Thus, it is possible that CORT could alter the gating and/or single channel conductance of NMDAR. Potentiated synaptic NMDAR function could be due to increased availability of cleft glutamate. Although the results of paired-pulse experiments do not support an increase in presynaptic glutamate release, CORT could nevertheless increase cleft glutamate levels by increasing glutamate release probability [12] or inhibiting glutamate uptake [60]. Increased cleft glutamate concentration might reasonably be expected to enhance both synaptic NMDAR and AMPAR functions, in opposition to our observations that CORT enhanced only NMDAR function. However, it is important to note that NMDAR has a much higher affinity for glutamate than AMPAR [61], and as such increased cleft glutamate concentration could have a larger impact on NMDAR function. Independent of potential cleft glutamate concentration increases, enhancement of NMDAR function could be mediated by phosphorylation of NMDAR [62], [63] subsequent to CORT-induced kinase activation. Further experiments are necessary to address the potential contributions of these mechanisms in enhancing synaptic NMDAR function.

The specific effect of CORT on NMDAR we observed contrasts with previous reports that CORT increased the surface expression [14], [15] and function of AMPAR [12], [13], [45]. Note that we detected no change in AMPAR-mediated synaptic function after CORT treatment in either field (Fig. 1) or whole cell recordings (Fig. 2). Moreover, surface biotinylation studies also confirmed no change in the expression of GluA1 subunit of AMPAR in adult hippocampal synapses (Fig. 4 and 5). The resolution of these discrepant findings may relate to differences in the examined temporal windows after CORT treatment between the present (0–2 hrs) and previous studies (>2 hrs). Indeed, most of these previous findings suggest CORT exerts a delayed-onset effect on AMPAR (but also see [12]). However, our findings do not rule out a potential impact of CORT on the trafficking of other AMPAR subunits, such as GluA2 whose mobility can be altered by stress [58] or CORT alone [14], [15].

An issue yet to be resolved is whether classical genomic effects of CORT underlie regulation of NMDAR function, especially with regards to slow onset changes. Indeed, expression profiling studies show that exposing hippocampal slices to the same concentration of CORT used in the present study induces changes in the expression of many plasticity-related genes [64]. Nonetheless, glutamate receptor function can also be regulated by nongenomic effects of CORT [12]. Such effects are likely mediated by membrane bound corticosteroid receptors and can be induced by CORT rendered membrane impermeable by covalent binding to albumin. In order to determine the contributions of genomic and nongenomic effects to CORT-induced regulation of NMDAR function, future studies should assess the effects of membrane impermeable CORT on NMDAR function. In addition, the influence of transcription inhibitors on CORT-induced regulation of NMDAR should be examined.

The timing of CORT treatment relative to plasticity induction is crucial for predicting the impact of this stress hormone on synaptic plasticity [8]. We report that while both LTP and LTD were facilitated during CORT exposure, facilitation of synaptic plasticity disappeared one hour after termination of CORT treatment. The temporally distinctive changes of NMDAR function after CORT treatment could underlie these time-dependent alterations in bidirectional synaptic plasticity. NMDAR mediates the induction of bidirectional synaptic plasticity and thus NMDAR-dependent LTP and LTD are likely facilitated during CORT treatment by the potentiation of synaptic NMDAR function. Our findings also suggest that the increase in GluN2A/GluN2B ratio observed one hour after CORT treatment is responsible for suppressing the early facilitation of bidirectional synaptic plasticity. LTD may require activation of GluN2B-containing NMDARs [33], [65] (but also see [66]), and an increase in the GluN2A/GluN2B ratio could abolish the facilitation of LTD by favoring GluN2A-containing receptor activation. While GluN2A-containing receptors could be important in LTP formation, such that pharmacologically blocking this receptor subpopulation [33], [65] or genetic truncation of the carboxyl terminal of this subunit suppresses LTP [67] (but also see [68], [69]), recent findings obtained from cultured slices suggest that excess GluN2A subunit also inhibits LTP formation [70]. It is hypothesized that the carboxyl tail of the GluN2A subunit could interact with signaling proteins that inhibit LTP, so that overexpressing exogenous GluN2A subunit suppresses LTP even when NMDAR function is potentiated. Our findings that CORT not only selectively increased endogenous GluN2A subunit expression in synapses, but also abolished the facilitation of LTP by CORT, support an inhibitory role of excess GluN2A subunit in LTP formation.

The short-term facilitation of bidirectional hippocampal synaptic plasticity by CORT could have important functional implications. While hippocampal LTP has been commonly regarded as a cellular mechanism of memory formation, recent findings suggest that hippocampal LTD is also necessary for certain hippocampus-dependent cognitive functions, such as spatial memory consolidation [65] and reversal learning [71], [72]. Facilitation of both hippocampal LTP and LTD by CORT could enhance encoding of threat-relevant information during stress. CORT-triggered slow-onset increase in GluN2A/GluN2B ratio could curtail the facilitation of synaptic plasticity. Whether the increased surface expression of GluN2A subunit serves a homeostatic role to maintain stability of the hippocampal network after the cessation of stressors remains to be determined.

One dilemma is how hippocampal neurons avoid excitotoxicity after CORT-induced facilitation of NMDAR, which could last for hours. Findings from pharmacological studies show that activation of GluN2B-containing NMDARs leads to excitotoxic cell death *in vivo* and *in vitro*, whereas activation of GluN2A-containing NMDARs is neuroprotective [73]. Moreover, GluN2B-containing NMDARs associate with pro-death cellular pathways [74], [75]. The slow-onset increase in neuroprotective GluN2A-containing receptors after CORT treatment could therefore safeguard the stressed brain against excitotoxicity.

The increase in GluN2A subunit after stress may also carry costs, potentially rendering the adult brain vulnerable to stress-related pathologies such as depression. For instance, preventing the phosphorylation of GluN2A at Y_1325_ residue, which is targeted by *src* to enhance NMDAR function, decreases the expression of depression-related behaviors of rodents in tail suspension and forced swim tests [76]. In addition, anxiety-related phenotypes in elevated plus maze and open field can be attenuated by genetic ablation of GluN2A [77]. Experiencing extreme and chronic stress could therefore pathologically enhance the expression of GluN2A subunit, which could mediate the association between major negative life events (e.g. death of a spouse or trauma) and the onset of depressive episodes in human adults [78]–[80]. Given that NMDAR antagonists exhibit promise as fast-acting antidepressants [81], [82], further investigations on the impact of CORT on GluN2A could be an important research avenue for revealing more effective and specific NMDAR-targeting antidepressants.

## Materials and Methods

### Ethics Statement

All care and use of animals was in accordance with the guidelines and policies of the Canadian Council on Animal Care to ameliorate suffering of animals in all work. This study (animal use protocol number #5935) was specifically approved by the Facility Animal Care Committee of Douglas Institute, McGill University.

### Materials

Unless otherwise specified, all materials were purchased from *Sigma-Aldrich*. CORT was initially dissolved in ethanol. The final concentration of ethanol in CORT-containing aCSF was 0.0007%. DNQX, dexamethasone and RU486 were dissolved in DMSO before preparing working concentrations of these drugs in aCSF containing 0.1% DMSO. Note that both basal AMPAR- and basal NMDAR-mediated synaptic responses were not affected by aCSF with these trace amount of organic solvents. Antibodies were purchased from *Sigma-Aldrich* (rabbit anti-GluN1, rabbit anti-actin), *Cell Signaling* (rabbit anti-GluN2A), *Novus* (rabbit anti-GluN2B) and *Chemicon* (rabbit anti-GluA1).

### Animals

Three-month-old adult male Sprague Dawley rats (*Charles River*) were housed in the Douglas Institute animal facility and maintained on a *12/12* light cycle with lights on at 08:00 am. Food and water were available *ad libitum*. Rats were housed at the facility for 5 days before electrophysiological experiments to reduce the influence of stress associated with transportation and housing in a novel environment. Finally, isoflurane anesthetized rats were decapitated during the nadir of their diurnal CORT rhythm (1–2 hours after lights on) to ensure they were euthanized under low endogenous levels of CORT (plasma CORT: 2.25±0.59 µg/dl (n = 9)).

### Slice preparation

Brains were rapidly removed from decapitated rats and coronal brain slices (400-µm thick) were cut in hyperosmotic, ice-cold and carbogenated (5% CO_2_, 95% O_2_) slice cutting solution (in mM: 252 sucrose, 2.5 KCl, 4 MgCl_2_, 0.1 CaCl_2_, 1.25 KH_2_PO_4_, 26 NaHCO_3_ and 10 glucose, ∼360 mOsmol/L) using a *Vibratome*. Freshly cut slices were incubated with carbogenated artificial cerebrospinal fluid (aCSF in mM: 125 NaCl, 2.5 KCl, 1 MgCl_2_, 2 CaCl_2_, 1.25 NaH_2_PO_4_, 26 NaHCO_3_ and 25 glucose, ∼310 mOsmol/L) at 32°C for 1 hour and subsequently maintained at room temperature. Bicuculline methobromide (10 µM) was used to block GABA_A_ receptor-mediated inhibitory synaptic transmission in all recordings. Postsynaptic responses were evoked by stimulating the Schaffer collateral-commissural pathway (constant current pulses (0.08 ms) through a tungsten bipolar electrode (*FHC*)) and recorded in hippocampal CA1 stratum radiatum. Synaptic responses were amplified and digitized by Multiclamp 700B and Digidata 1400 respectively (*Axon*), and stored in a PC for offline analysis using Clampfit (*Axon*). All recordings were performed at room temperature.

### Field and whole-cell recording

Postsynaptic responses were evoked at 0.05 Hz before and after the induction of synaptic plasticity. Field excitatory postsynaptic potentials (fEPSPs) were detected by aCSF-filled glass electrodes. To isolate NMDAR-mediated fEPSPs (NMDAR-fEPSP), field recording was performed in the presence of low Mg^2+^ (0.05 mM) aCSF with an AMPAR antagonist DNQX (20 µM). Isolated NMDAR-fEPSP can be abolished by an NMDAR antagonist APV (50 µM, Fig. 1). Evoked excitatory postsynaptic currents (EPSCs) were recorded in the whole-cell mode using patch pipettes containing (mM) 110 Cs-gluconate, 17.5 CsCl, 2 MgCl_2_, 0.5 EGTA, 10 HEPES, 4 ATP, and 5 QX-314 (*Alomone Labs*), with pH adjusted to 7.2 by CsOH (∼290 mOsmol/L).

### Biochemical fractionation of biotinylated synaptosomal membranes

We investigated changes in surface expression of synaptic glutamate receptor caused by CORT treatment using a modified biotinylation assay. Biotinylating reagent has been shown to penetrate hundreds of µm through brain slices [83]. However, we found that incubating slices at room temperature for recovery and drug treatment greatly increased the labeling of intracellular proteins by biotinylating reagent, perhaps due to exacerbated tissue damage of the cut surface of slices caused by hours of room temperature incubation. To reduce labeling of intracellular proteins caused by tissue damage, we performed drug treatments on intact hippocampi (Fig. 4 and 5). Intact hippocampal preparation has been shown to remain viable in standard electrophysiological conditions and exhibit network activities such as theta and gamma rhythms for many hours [84]. Briefly, intact hippocampi were dissected from adult rat brains and incubated in carbogenated aCSF for 2 hours at room temperature before CORT treatment. The left and right hippocampi from each rat were assigned to either control or CORT-treated conditions. Hippocampal side was counter-balanced to ensure similar numbers of left and right hippocampi in both the treatment and control groups. Hippocampi from each rat were treated with either CORT (100 nM) or aCSF (control) for 30 min. Either immediately or one hour after treatment, both control and CORT-treated hippocampi were cut into 400 µm slices using a tissue chopper and immediately biotinylated to label surface membrane proteins at 4°C. Note that since slices were biotinylated immediately after cutting, we did not see significant labeling of intracellular proteins, such as actin, by biotinylating reagent in our samples (see Fig. 4 and 5).

Freshly cut slices from control and CORT treated hippocampi were incubated with cold HEPES-buffered aCSF (H-aCSF) containing 1 mg/ml EZ-Link Sulfo-NHS-Biotin (*Pierce*) on ice with mild agitation for 30 min. Unbound biotin was removed by two 5-min washes of cold 100 mM glycine in H-aCSF on ice. After two more 5-min washes with cold H-aCSF without glycine, slices were homogenized to extract synaptosomal membranes [85]. This protocol allowed us to examine changes in glutamate receptor expression in the synaptosomal fraction of hippocampal lysates, which is closely related to electrophysiological measures observed in hippocampal synapses. Briefly, slices were homogenized in lysis buffer (in mM: 10 Tris base, 5 NaF, 1 Na_3_VO_4_, 1 EDTA, 1 EGTA, pH 7.4) with 320 mM sucrose. Homogenate was centrifuged for 10 min at 800×g, 4°C and the supernatant was centrifuged at 9200×g for 15 min at 4°C. The resulting P2 pellet was resuspended in lysis buffer containing 35.6 mM sucrose and incubated on ice for 30 min. Resuspended P2 was then centrifuged at 25,000×g for 30 min at 4°C to obtain the LP1 pellet, which contains synaptosomal membranes. LP1 pellet was resuspended in lysis buffer containing 1% SDS and sonicated using a probe sonicator. After centrifuging at 15,000×g for 5 min, the pellet was discarded and the supernatant, containing solubilized synaptosomal proteins, retained. Protein concentration was determined by the Bradford method (*BioRad*) and synaptosomal proteins were used for estimating surface expression of glutamate receptors in synapses.

### Estimating the surface expression of synaptic glutamate receptors

To estimate the surface expression of glutamate receptors in hippocampal synapses, we calculated the ratio of biotinylated synaptosomal proteins, which represent synaptic proteins on cell surface, vs. total synaptosomal proteins using western blotting. To purify biotinylated proteins, a fixed amount of synaptosomal proteins from each rat was incubated with avidin-agarose at 4°C for 30 min. Excess avidin-agarose was used to ensure all biotinylated proteins were pulled down. Both surface and total proteins from each rat were separated by SDS-PAGE and probed for GluN1 (1∶1000), GluN2A (1∶1000), GluN2B (1∶1000), GluA1 (1∶500), and actin (1∶1000). Surface expression of glutamate receptors was expressed as the ratio of the band intensity of surface to total protein on western blots.

### Statistical analysis

All data are presented as mean±SEM. *Student*'*s t test* was used for comparisons between 2 groups. The surface expression of glutamate receptor in control and CORT-treated hippocampi from each rat was compared using paired *Student*'*s t test*. Multiple-group comparisons were analyzed using *ANOVA* (simple or repeated measures) followed by post hoc *Fisher*'*s test* for pairwise comparisons.
